# Mammalian Comparative Sequence Analysis of the *Agrp* Locus

**DOI:** 10.1371/journal.pone.0000702

**Published:** 2007-08-08

**Authors:** Christopher B. Kaelin, Gregory M. Cooper, Arend Sidow, Gregory S. Barsh

**Affiliations:** 1 Department of Genetics, Stanford University, Stanford, California, United States of America; 2 Department of Genome Sciences, University of Washington, Seattle, Washington, United States of America; 3 Department of Pathology, Stanford University, Stanford, California, United States of America; 4 Department of Pediatrics, Stanford University, Stanford, California, United States of America; University of Sevilla, Spain

## Abstract

*Agouti-related protein* encodes a neuropeptide that stimulates food intake. *Agrp* expression in the brain is restricted to neurons in the arcuate nucleus of the hypothalamus and is elevated by states of negative energy balance. The molecular mechanisms underlying *Agrp* regulation, however, remain poorly defined. Using a combination of transgenic and comparative sequence analysis, we have previously identified a 760 bp conserved region upstream of *Agrp* which contains STAT binding elements that participate in *Agrp* transcriptional regulation. In this study, we attempt to improve the specificity for detecting conserved elements in this region by comparing genomic sequences from 10 mammalian species. Our analysis reveals a symmetrical organization of conserved sequences upstream of *Agrp*, which cluster into two inverted repeat elements. Conserved sequences within these elements suggest a role for homeodomain proteins in the regulation of *Agrp* and provide additional targets for functional evaluation.

## Introduction

AGRP is an orexigenic, hypothalamic peptide whose role in energy homeostasis has been conserved during vertebrate evolution. In a wide range of species, including mammals [1], [2], birds [3], [4], and fish [5], neuronal *Agrp* expression is restricted to a discrete population of neurons that sense the levels of peripheral energy stores, and is dramatically elevated by deficits in energy balance.

AGRP neurons directly receive information about energy stores from leptin, an adipocyte-derived hormone that circulates at levels proportional to fat mass. Diminished levels of circulating leptin correspond to increased *Agrp* mRNA levels [6]. Leptin receptor occupancy on AGRP neurons results in the phosphorylation of two transcription factors, FoxO1 [7] and STAT3 [8], inducing the cytoplasmic localization of FoxO1 and the nuclear localization of STAT3. A recent study by Kitamura and colleagues [7] implicates a direct, reciprocal role for both STAT3 and FoxO1 in *Agrp* regulation, where STAT3 represses and FoxO1 stimulates *Agrp* transcription. However, conserved STAT binding sites in the *Agrp* promoter region do not function as simple repressor elements and, paradoxically, are required for fasting induced stimulation of *Agrp* transcription [9], suggesting a more complex mechanism of regulation involving additional cofactors.

Support for this also stems from the observation that peripheral energy signals other than leptin regulate *Agrp* expression. For example, AGRP neurons directly mediate the orexigenic effects of glucocorticoids [10], [11] and ghrelin [12]. Central administration of either elevates *Agrp* mRNA levels [13], [14], and intact glucocorticoid signaling is required for fasting induced increases in *Agrp* expression [14]. Neither STAT3 nor FoxO1 is a known target of glucocorticoid or ghrelin signaling.

Cross-species comparative sequence analysis has facilitated the detection of functional elements that participate in transcriptional regulation [15]. Previously, we identified mouse BAC clones that recapitulate *Agrp* expression in transgenic mice and that contain regions of high sequence conservation between mouse and human, including a 760 bp region located immediately upstream of *Agrp* [16]. While we could identify candidate binding elements in this region based on biological inference [9], the level of resolution provided by mouse/human sequence comparison did not permit us to predict putative binding elements based solely on sequence conservation.

The inclusion of sequences from multiple, divergent species sharing a commonly derived phenotype improves the resolution of comparative sequence analysis. The conserved nature of *Agrp* expression in vertebrates suggests that sequence comparisons of disparate vertebrate species provide an appropriate evolutionary scope for identifying *Agrp* regulatory elements. However, *Agrp* genomic sequences from distantly related vertebrate species have diverged to the extent that regional conservation is no longer detectable using traditional alignment methodologies [9]. Here, we describe a comparative sequence analysis of an *Agrp* genomic region from ten mammalian species, representing several different orders and all three subclasses of mammalia. Our analysis reveals a symmetrical organization of conserved sequences upstream of *Agrp*, which cluster into two inverted repeat elements (IREs). The proximity of the elements to *Agrp* and the nearly perfect evolutionary conservation of their specific constituent sequences in all ten mammalian species and in chickens suggest a role in *Agrp* regulation. In addition, the resolution of the conserved elements provided by this approach allows general predictions concerning putative trans-regulatory factors.

## Methods

### Matrix comparisons of *Agrp* genomic regions

Genomic sequence surrounding the *Agrp* locus for chimpanzee (*Pan troglodytes*), human (*Homo sapiens*), dog (*Canis familiaris*), mouse (*Mus musculus*), and rat (*Rattus norvegicus*) was obtained from publicly available genome assemblies (chimpanzee assembly CHIMP1, human assembly 35, dog assembly CanFam1.0, mouse assembly m33, rat assembly RGSC 3.4, respectively) on the UCSC genome browser (http://genome.ucsc.edu). Genomic sequence for other species, including chicken (*Gallus gallus*), cat (*Felis catus*), cow (*Bos taurus*), tenrec (*Echinops telfairi*), platypus (*Ornithorhynchus anatinus*), and opossum (*Monodelphis domestica*), was obtained from the trace sequence archives at NCBI (http://www.ncbi.nlm.nih.gov) and assembled using SeqMan (DNAStar, Inc., Madison, WI). Our assembly of chicken genomic sequence at the *Agrp* locus differed from the publicly available genome assembly (Chicken v1.0), since additional trace sequence was available. Notably, the chicken sequence corresponding to the STAT site and inverted repeat element is not present in the public assembly. Our assembly of the chicken *Agrp* locus is provided as supplementary material (**Text S1**). Matrix comparisons were done for each species pair using the dot plot feature in the MegAlign software package (DNAStar, Inc., Madison, WI). We empirically determined an appropriate visualization threshold for dot plots after testing several window sizes and sequence identity levels. The threshold of 70% sequence identity over 30 bp provides specificity to detect relatively small, highly conserved sequences while excluding the majority of background signal.

### Measurement of evolutionary constraint

Approximately 1.8 kb of genomic sequence from the *Agrp* locus of 10 mammalian species, including the transcription unit and the proximal conserved region, were aligned using the multi-Lagan alignment tool (http://lagan.stanford.edu) [17]. All sequences used in this analysis are provided as supplementary material (**Text S2**). Because the function of putative *Agrp* regulatory sequences has been previously interrogated using mouse transgenic models [9], we chose to use mouse sequence (corresponding to mouse Chromosome 8:104864049–104863970 from the mouse genome Build 34) as a reference for analysis. To maintain consistency between mouse genome assembly and our alignment coordinates, the multi-species alignment was compressed such that the mouse sequence is ungapped. We measured evolutionary constraint at each nucleotide position using Genomic Evolutionary Rate Profiling (GERP), the methodology of which is described in detail by Cooper et al. [18]. Briefly, GERP estimates an expected neutral substitution rate for a group of aligned species, and quantifies deviation from the estimated neutral rate for each column in a species alignment. The sum of deviation scores in consecutively constrained alignment columns provides a metric of evolutionary constraint, termed a rejected substitution (RS) score. Therefore, the RS score associated with a constrained region is impacted by both the magnitude of deviation from the estimated neutral rates at each column position and the number of consecutively constrained columns. Since some functional regions contain unconstrained nucleotides, GERP allows for RS scores to be merged across unconstrained alignment columns. In this analysis, RS scores were merged across a single unconstrained column. The neutral rate estimate for species in our alignment, based on previous estimates from other genomic loci [18], [19], is 2.75 substitutions/site. The maximum false positive score, based on ungapped alignment segments from permuted versions of the data set, was 13.6±0.8 (n = 10). **Table 1** summarizes the RS scores for this region ranking above the maximum false positive estimates for permuted data sets [18], all of which are independent of sequence annotation. The results of GERP analysis were visualized using the java-based graphical user interface, Application for Browsing Constraints (ABC) [20].

**Table 1:** Constrained elements at the *Agrp* locus identified by GERP.

RS score^a^	Start Position^b^	End Position^c^	Length^d^	Overlapping Region
234.0	+563	+710	147 bp	Exon 4
67.1	−832	−792	40 bp	IRE A
64.2	−8	+46	54 bp	Exon2
61.4	−472	−428	44 bp	IRE B
32.1	−755	−731	24 bp	STATA
26.8	−179	−167	13 bp	TATA
26.2	−527	−515	12 bp	STAT B
21.8	+306	+335	29 bp	Exon 3
17.6	+546	+560	14 bp	Exon 4
16.3	−363	−348	15 bp	None
13.9	+283	+297	14 bp	Exon 3

a The Rejected Substitution score is a measurement of evolutionary constraint, based on Genomic Evolutionary Rate Profiling [18]. The table includes a list of scores above the maximum false positive score for this data set (see methods).

b,c The initial and terminal columns within the sequence alignment defining the region of evolutionary constraint (coordinates based on mouse genomic sequence, relative to the translational start site of *Agrp*).

d Length of evolutionarily constrained region defined as the sum of consecutive alignment columns with positive RS scores.

In order to calculate constraint scores for *Agrp* exons, the STAT sites, and the IREs, we modified the concept of an RS score, such that the score for constraint represents the sum of constrained alignment columns in these regions while ignoring unconstrained alignment columns. In this way, the constraint score reflects the level of sequence conservation within a particular region without penalizing unconstrained positions (**Table 2**).

**Table 2:** Constrained regions at the *Agrp* locus.

Element	Length (bp)	Constraint (RS score^a^)	Constraint/length	% Constrained Positions
IRE A	45	75.5	1.68	37/45 (82%)
STAT A	9	24.8	2.76	9/9 (100%)
STAT B	9	23.1	2.57	9/9 (100%)
IRE B	49	77.8	1.59	44/49 (90%)
Exon 1	31	20.3	0.87	16/31 (52%)
Exon 2	133	130.4	1.20	90/133 (68%)
Exon 3	83	77.1	1.15	54/83 (65%)
Exon 4	223	295.3	1.54	160/223 (72%)

a Modified Rejected Substitution score calculated using Genomic Evolutionary Rate Profiling (see methods), based on the alignment of 10 mammalian species.

## Results

**Figure 1:**
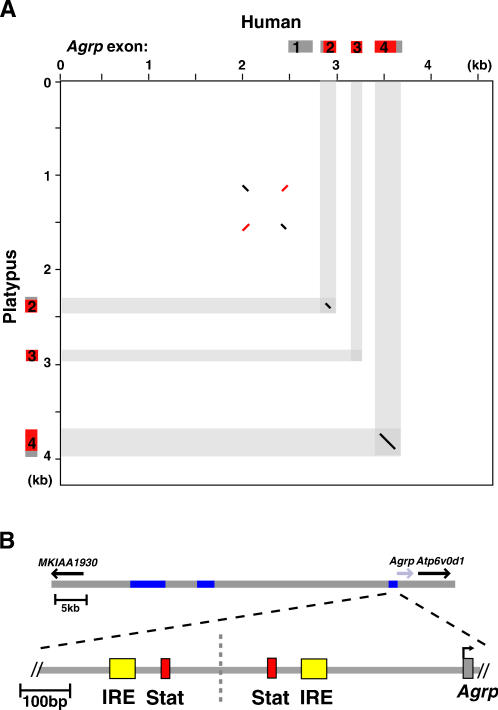
Conserved inverted repeat elements upstream of *Agrp*. (A) Matrix comparison of human (X-axis) and platypus (Y-axis) genomic sequences at the *Agrp* locus with a stringency threshold set at 70% identity over a 30 bp window reveals conservation in *Agrp* coding exons (shaded areas) and in the upstream region, representative of similar comparisons between distantly related mammalian species. For reference, the position of *Agrp* exonic sequence is shown on either axis, with coding sequence in red and non-coding sequence in grey. Sequence similarity between forward and reverse strands is plotted in black and red, respectively. (B) Shows the genomic locus containing *Agrp* and neighboring genes, *MKIAA1930* and *Atp6v0d1*. The blue boxes indicate areas of mouse/human sequence conservation located within a functionally defined *Agrp* regulatory region [16]. The relative position of the conserved IREs (in yellow) and the STAT binding sites (in red) upstream of *Agrp* are illustrated below. Sequence similarity and the symmetrical structure of the elements reveal an ancient duplication within the *Agrp* region, the axis of which is indicated by a vertical, dashed line.

Using a matrix analysis that did not rely on global sequence alignment, we searched for short, nearly identical sequences (30 bp of 70% sequence identity) surrounding *Agrp* in pairwise comparisons of vertebrate species. Analysis of distantly related mammalian species, such as human and platypus (**Figure 1A**), identified two highly conserved regions, each extending ∼40 bp. Notably, the detection threshold of these regions, which we termed IREs for reasons described below, exceeds the detection threshold for some *Agrp* exons when distantly related mammals are compared (**Figure 1A**). IREs were not detected using matrix analysis when pairwise comparisons were extended to include non-mammalian vertebrate species (data not shown).

As shown in **Figure 1B**, the IREs are located immediately upstream of *Agrp*, each one flanking a conserved STAT binding element [7], [9]. Moreover, the IREs show striking similarity to one another when aligned in a reverse complementary manner (**Figure 1A**). The sequence similarity between the IREs and their symmetrical orientation with respect to the STAT binding elements suggests that the genomic region upstream of *Agrp* underwent a duplication that occurred prior to mammalian radiation, and portions of the duplicated region have been subjected to purifying selection such that specific sequences (those corresponding to the STAT elements and IREs) are maintained in two closely related copies.

**Figure 2:**
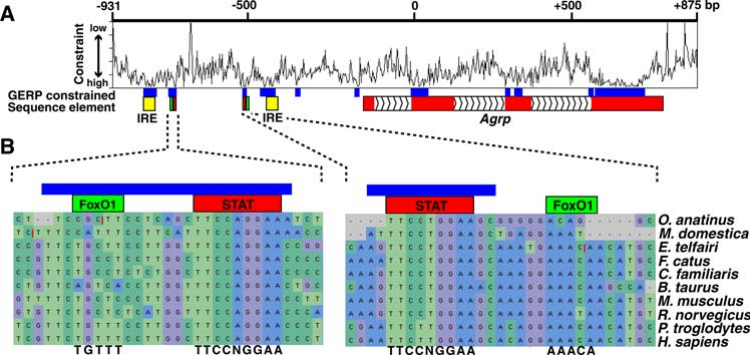
GERP constraint plot for the *Agrp* genomic locus. (A) A plot illustrating evolutionary constraint variation, measured by GERP, within a 1.8 kbp alignment of 10 mammalian species across the *Agrp* locus. The RS scores associated with significantly constrained regions (shown as blue boxes) are reported in Table 1. Coordinates refer to the position in mouse genomic sequence relative to the first position of the *Agrp* translational start site. While GERP analysis is independent of sequence annotation, constrained sequences overlap with the IREs (yellow), putative STAT and FoxO1 binding elements (red and green, respectively), and *Agrp* coding regions (red). (B) Sequence alignments indicating the relative conservation of putative FoxO1 and STAT binding sites, with consensus sites shown below the alignments.

To quantify evolutionary constraint for different conserved regions at the *Agrp* locus, we aligned 1.8 kb of *Agrp* genomic sequence from 10 mammalian species – human, chimpanzee, mouse, rat, cow, dog, cat, tenrec, opossum, and platypus - with multi-lagan. We then used GERP [18] to estimate evolutionary constraint across the sequence alignment. **Figure 2A** shows constraint variation across the *Agrp* locus and compares the position of constrained sequences with annotated elements. Based on this approach, the level of evolutionary constraint for sequences overlapping the IREs and the STAT binding sites are extremely high relative to other genomic regions [18] and well above false positive estimates (**Table 1** and methods). In fact, using the average constraint score per base pair as a metric, the IREs and STAT binding sites are more conserved than *Agrp* coding sequences in this alignment of species (**Table 2**). In contrast, putative FoxO1 binding sites, located adjacent to the STAT sites, are not well-conserved (**Figure 2B**).

**Figure 3:**
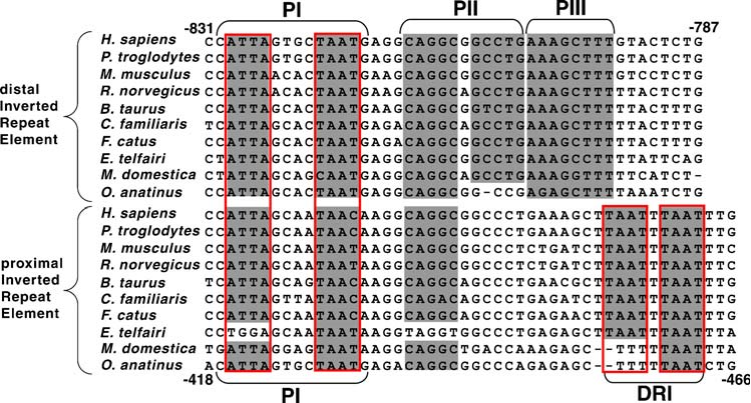
Mammalian alignment of the inverted repeat element. Sequences comprising the inverted repeat elements from 10 mammalian species were aligned using the multi-Lagan tool. To demonstrate their sequence homology, distal IRE and proximal IRE are shown in a forward and reverse orientation, respectively. Both IREs contain multiple conserved sequence motifs, including palindromic (PI-PIII) and direct (DRI) repeat sequences (shaded in grey), which likely function as transcription factor binding modules. Putative homeodomain transcription factor binding motifs are outlined in red.

A remarkable aspect of the IREs, in addition to their evolutionary constraint, is their organization into palindromic sequence blocks. As illustrated in **Figure 3**, the distal IRE contains 3 conserved palindromic motifs, constituting 31 of 39 constrained nucleotide positions. The proximal IRE also contains a conserved palindromic motif and a direct repeat motif, constituting 18 of 39 constrained nucleotide positions, as well as conserved half sites for palindromic motifs identified in the distal IRE (**Figure 3**). Since palindromes commonly serve as recognition sequences for transcription factors, the clustering of conserved palindromic sequences suggests that the IREs may function as composite binding modules for a complex of transcription factors.

**Figure 4:**
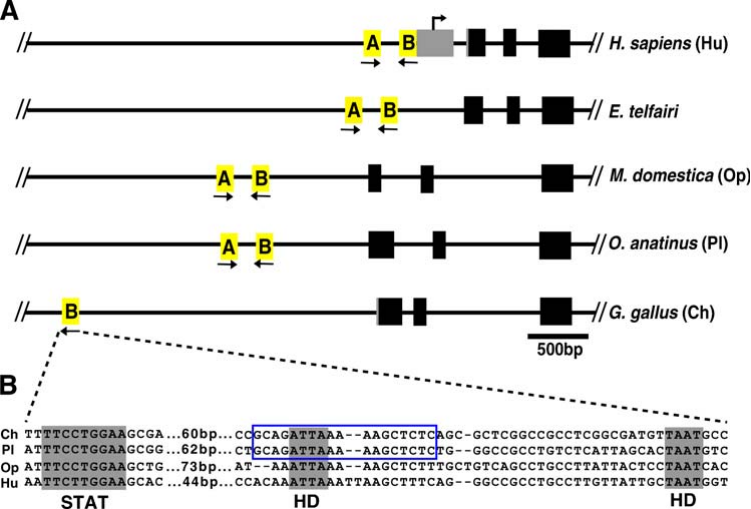
Genomic structure of the *Agrp* locus in divergent vertebrate species. (A) The top panel illustrates the relative position of exons (coding regions and UTRs are represented in black and grey, respectively) and IREs (in yellow) at the *Agrp* locus in humans (*H. sapiens*, Hu), tenrec (*E. telfairi*), opossum (*M. domestica*, Op), platypus (*O. anatinus*, Pl) and chicken (*G. gallus*, Ch). The transcriptional start position has only been mapped for human *Agrp*, as indicated. The orientation of the IREs with respect to each other is indicated with black arrows. Notably, the position of the regulatory region(s) relative to the transcription unit varies in different species. (B) A sequence alignment of the STAT site and IRE, in chicken (ch), platypus (pl), opossum (op), and human (hu), which comprises the region represented by the proximal IRE, “B”, shown in (A). Sequences conserved between chicken and mammalian species are shaded in grey and include the STAT binding element and two putative homeodomain binding motifs (HD). Also, an 18 bp sequence within the IRE is perfectly conserved between chicken and platypus (outlined in blue).

We could not confidently align orthologous sequence from non-mammalian species, such as chicken and zebrafish, using multi-lagan. Instead, we scanned genomic regions upstream of *Agrp* in these species, using matrix analysis, to detect motifs similar to those conserved in mammalian IREs. As shown in **Figure 4**, we identified a region located ∼2.5 kb upstream of the first *Agrp* coding exon in chicken that demonstrates similarity to the proximal IRE of mammals and contains some of the conserved motifs common to both mammalian IREs. In particular, the chicken IRE-like region includes an 18 bp segment which is perfectly conserved with the proximal platypus IRE (**Figure 4B**), a striking observation considering the extensive evolutionary distance between these species (∼300 million years). Moreover, a consensus STAT site is located 60 bp upstream of the chicken IRE-like region, indicating that the positional relationship of these elements is also preserved (**Figure 4B**). We were unable to identify a second region in chicken genomic sequences corresponding to the distal IRE and STAT binding element, and we were also unable to identify similar elements near *Agrp* in sequenced teleost fish species.

## Discussion

In this study, we leveraged the recent availability of sequence information from different species and new methodologies in sequence comparison to improve the ability to detect conserved sequences at the *Agrp* locus. The resolution provided by comparing 10 mammalian sequences with GERP reveals a previously unappreciated structure to the region upstream of *Agrp*. Conservation of this genomic structure in mammals indicates that a duplication event occurred prior to mammalian speciation in which the highly conserved sequences have been subsequently maintained in two homologous copies. Nevertheless, orthologous IREs, even from distantly related species such as human and platypus, are more similar than paralogous IREs, suggesting that paralogous IREs have acquired different functions that are preserved in mammals.

*Agrp* is also expressed prominently in the adrenal gland [1], [21], where it is thought to regulate steroidogenesis [22]. In both the arcuate nucleus and the adrenal gland, *Agrp* expression levels are elevated by food deprivation and leptin deficiency [1], [21], suggesting a common mechanism of regulation. However, the transcript lengths in these expression sites differ. In mice and humans, hypothalamic transcripts have a longer 5′ untranslated region than adrenal transcripts, indicating alternative transcriptional start sites [1]. Notably, the difference in transcript length, ∼300 bp, is similar to the distance between the IREs. It is therefore plausible to speculate that different IREs may regulate *Agrp* expression in different expression sites.

We also identified a region upstream of chicken *Agrp* containing both a STAT site and an IRE-like element, which are strikingly similar to their mammalian counterparts in spacing and composition. The chicken STAT site is perfectly conserved with mammalian STAT sites; the IRE-like element, while less conserved, retains some of the features of mammalian IREs, including the TAAT motifs which display the highest degree of constraint in mammals (**Figure 3**). If the IREs function as composite binding modules for multiple transcription factors, then the chicken IRE-like element might contain a subset of the binding modules found in mammalian IREs as well as distinct modules. An analogous comparison of orthologous regions in other avian species may reveal a pattern of conserved sequences within these modules that is different from mammals, reflecting differences in *Agrp* regulation, and could potentially provide a molecular perspective for the evolutionary development of pathways regulating *Agrp* transcription.

The TAAT sequences comprising the core regions of conserved palindromes and direct repeats in both IREs are canonical binding sites for homeodomain (HD) transcription factors, which are generally involved in the regulation of development and cell fate but also function post-developmentally to modulate cell type specific gene expression. While HD transcription factors represent a large family of proteins with over 100 members, most display restricted expression patterns that are predictive of function. Several HD transcription factors are regionally expressed in the hypothalamus.

HD transcription factors have been previously implicated in the regulation of neuropeptide gene expression, and conserved HD binding sites have been identified in the regulatory regions of other hypothalamic neuropeptide genes, including *Pomc* [23], *Orexin* [24], and *Gonadotropin Releasing Hormone* (*GnRH*) [25]. In particular, the transcriptional regulation of *GnRH* has been extensively studied, due in part to an immortalized cell line which has retained important characteristics of endogenous GnRH neurons. Multiple HD transcription factors cooperatively bind to the sequences upstream of *GnRH*, regulating not only cell type specific expression but also dynamic, physiologic responses by serving as scaffolds for additional cofactors [26], [27]. The studies of GnRH regulation offer an appealing framework for extending the model of *Agrp* regulation proposed by Kitamura and colleagues to integrate observations regarding input from other signals and the organization of conserved sequences identified in this analysis.

Within the 760 bp region upstream of *Agrp* defined by mouse/human sequence conservation, the IREs and STAT binding sites comprise the majority of non-coding sequence conserved among mammals. Only two other non-coding sequences, which are both ∼15 bp in length, meet the threshold for constraint set by GERP (**Table 1**). One of these overlaps with a putative TATA box and likely binds the core transcriptional apparatus. The second, which is located between the putative TATA box and the proximal IRE, barely meets the significance threshold and does not contain obvious candidate binding sites.

In contrast to the STAT binding sites, the putative FoxO1 binding sites identified by Kitamura and colleagues, which are located between to the IREs and STAT binding sites, are not well-conserved (**Figure 2B**). Nevertheless, injection of an adenovirus encoding a constitutively active FoxO1 in mice increases *Agrp* expression and stimulates food intake, and chromatin immuno-precipitation studies confirm that FoxO1 binds to this region. These observations suggest that (1) FoxO1 is capable of recognizing non-canonical FoxO1 binding sites in some species, (2) FoxO1 regulates *Agrp* expression only in a subset of mammalian species, (3) or FoxO1 associates with *Agrp* upstream regions through a mechanism that does not involve a direct interaction with the putative FoxO1 binding sites. Notably, direct interactions between forkhead and HD transcription factors have been previously described and implicated as a general mechanism for post-developmental regulation of gene expression [28], [29].

Like AGRP, NPY is a hypothalamic neuropeptide that stimulates food intake when administered in the CNS [30]. *Agrp* and *Npy* are expressed by the same population of neurons in the arcuate nucleus of the hypothalamus and their expression co-varies in response to a number of stimuli [6], suggesting that similar cis-regulatory modules may control *Agrp* and *Npy* expression. In neuronal cell lines, HD transcription factors bind to TAAT motifs located within a regulatory region identified upstream of *Npy* [31]. Comparing the organization of conserved non-coding sequences at these two loci might reveal both common and disparate mechanisms of transcriptional regulation. Given the functional similarity between *Agrp* and *Npy*, the extent to which their regulatory mechanisms overlap may define distinct roles for these neuropeptides in energy homeostasis.

From a medical standpoint, the significance of understanding *Agrp* transcriptional regulation stems from the observation that common obesity is characterized by a blunted central response to peripheral energy signals, a phenomenon referred to as leptin resistance [32]. The mechanisms underlying leptin resistance remain poorly understood, but clearly result in the disregulation of neuropeptide genes, including *Agrp* [33]. The transcriptional regulation of *Agrp* is likely to involve the integration of information from several inputs. The nature of conserved sequences upstream of *Agrp* suggests a model whereby homeodomain proteins participate in the process of integrating these signals. The IREs identified in this analysis provide genomic targets for evaluating the role of specific transcription factors in the regulation of *Agrp* expression.

## Supporting Information

Text S1 A FASTA file containing 6144 bp of genomic sequence corresponding to the chicken Agrp locus.
(0.01 MB TXT)

Text S2 A FASTA file containing the mammalian sequences used for GERP analysis.
(0.02 MB TXT)

## References

[1] Shutter JR, Graham M, Kinsey AC, Scully S, Luthy R (1997). Hypothalamic expression of ART, a novel gene related to agouti, is up-regulated in obese and diabetic mutant mice.. Genes Dev.

[2] Henry BA, Rao A, Ikenasio BA, Mountjoy KG, Tilbrook AJ (2001). Differential expression of cocaine- and amphetamine-regulated transcript and agouti related-protein in chronically food-restricted sheep.. Brain Res.

[3] Boswell T, Li Q, Takeuchi S (2002). Neurons expressing neuropeptide Y mRNA in the infundibular hypothalamus of Japanese quail are activated by fasting and co-express agouti-related protein mRNA.. Brain Res Mol Brain Res.

[4] Phillips-Singh D, Li Q, Takeuchi S, Ohkubo T, Sharp PJ (2003). Fasting differentially regulates expression of agouti-related peptide, pro-opiomelanocortin, prepro-orexin, and vasoactive intestinal polypeptide mRNAs in the hypothalamus of Japanese quail.. Cell Tissue Res.

[5] Cerda-Reverter JM, Peter RE (2003). Endogenous melanocortin antagonist in fish: structure, brain mapping, and regulation by fasting of the goldfish agouti-related protein gene.. Endocrinology.

[6] Schwartz MW, Woods SC, Porte D, Seeley RJ, Baskin DG (2000). Central nervous system control of food intake.. Nature.

[7] Kitamura T, Feng Y, Kitamura YI, Chua SC, Xu AW (2006). Forkhead protein FoxO1 mediates Agrp-dependent effects of leptin on food intake.. Nat Med.

[8] Vaisse C, Halaas JL, Horvath CM, Darnell JE, Stoffel M (1996). Leptin activation of Stat3 in the hypothalamus of wild-type and ob/ob mice but not db/db mice.. Nat Genet.

[9] Kaelin CB, Gong L, Xu AW, Yao F, Hockman K (2006). Signal transducer and activator of transcription (stat) binding sites but not stat3 are required for fasting-induced transcription of agouti-related protein messenger ribonucleic acid.. Mol Endocrinol.

[10] Drazen DL, Wortman MD, Schwartz MW, Clegg DJ, van Dijk G (2003). Adrenalectomy alters the sensitivity of the central nervous system melanocortin system.. Diabetes.

[11] Hisano S, Kagotani Y, Tsuruo Y, Daikoku S, Chihara K (1988). Localization of glucocorticoid receptor in neuropeptide Y-containing neurons in the arcuate nucleus of the rat hypothalamus.. Neurosci Lett.

[12] Chen HY, Trumbauer ME, Chen AS, Weingarth DT, Adams JR (2004). Orexigenic action of peripheral ghrelin is mediated by neuropeptide Y and agouti-related protein.. Endocrinology.

[13] Kamegai J, Tamura H, Shimizu T, Ishii S, Sugihara H (2000). Central effect of ghrelin, an endogenous growth hormone secretagogue, on hypothalamic peptide gene expression.. Endocrinology.

[14] Makimura H, Mizuno TM, Isoda F, Beasley J, Silverstein JH (2003). Role of glucocorticoids in mediating effects of fasting and diabetes on hypothalamic gene expression.. BMC Physiol.

[15] Pennacchio LA, Rubin EM (2001). Genomic strategies to identify mammalian regulatory sequences.. Nat Rev Genet.

[16] Kaelin CB, Xu AW, Lu XY, Barsh GS (2004). Transcriptional regulation of agouti-related protein (Agrp) in transgenic mice.. Endocrinology.

[17] Brudno M, Do CB, Cooper GM, Kim MF, Davydov E (2003). LAGAN and Multi-LAGAN: efficient tools for large-scale multiple alignment of genomic DNA.. Genome Res.

[18] Cooper GM, Stone EA, Asimenos G, Green ED, Batzoglou S (2005). Distribution and intensity of constraint in mammalian genomic sequence.. Genome Res.

[19] Cooper GM, Brudno M, Green ED, Batzoglou S, Sidow A (2003). Quantitative estimates of sequence divergence for comparative analyses of mammalian genomes.. Genome Res.

[20] Cooper GM, Singaravelu SA, Sidow A (2004). ABC: software for interactive browsing of genomic multiple sequence alignment data.. BMC Bioinformatics.

[21] Ollmann MM, Wilson BD, Yang YK, Kerns JA, Chen Y (1997). Antagonism of central melanocortin receptors in vitro and in vivo by agouti-related protein.. Science.

[22] Doghman M, Soltani Y, Rebuffet V, Naville D, Begeot M (2007). Role of Agouti-related protein in adrenal steroidogenesis.. Mol Cell Endocrinol.

[23] de Souza FS, Santangelo AM, Bumaschny V, Avale ME, Smart JL (2005). Identification of neuronal enhancers of the proopiomelanocortin gene by transgenic mouse analysis and phylogenetic footprinting.. Mol Cell Biol.

[24] Moriguchi T, Sakurai T, Takahashi S, Goto K, Yamamoto M (2002). The human prepro-orexin gene regulatory region that activates gene expression in the lateral region and represses it in the medial regions of the hypothalamus.. J Biol Chem.

[25] Kelley CG, Givens ML, Rave-Harel N, Nelson SB, Anderson S (2002). Neuron-restricted expression of the rat gonadotropin-releasing hormone gene is conferred by a cell-specific protein complex that binds repeated CAATT elements.. Mol Endocrinol.

[26] Rave-Harel N, Miller NL, Givens ML, Mellon PL (2005). The groucho-related gene family regulates the gonadotropin-releasing hormone gene through interaction with the homeodomain proteins MSX1 and OCT1.. J Biol Chem.

[27] Clark ME, Mellon PL (1995). The POU homeodomain transcription factor Oct-1 is essential for activity of the gonadotropin-releasing hormone neuron-specific enhancer.. Mol Cell Biol.

[28] Foucher I, Montesinos ML, Volovitch M, Prochiantz A, Trembleau A (2003). Joint regulation of the MAP1B promoter by HNF3beta/Foxa2 and Engrailed is the result of a highly conserved mechanism for direct interaction of homeoproteins and Fox transcription factors.. Development.

[29] Foucher I, Volovitch M, Frain M, Kim JJ, Souberbielle JC (2002). Hoxa5 overexpression correlates with IGFBP1 upregulation and postnatal dwarfism: evidence for an interaction between Hoxa5 and Forkhead box transcription factors.. Development.

[30] Stanley BG, Kyrkouli SE, Lampert S, Leibowitz SF (1986). Neuropeptide Y chronically injected into the hypothalamus: a powerful neurochemical inducer of hyperphagia and obesity.. Peptides.

[31] Mayer CM, Cai F, Cui H, Gillespie JM, MacMillan M (2003). Analysis of a repressor region in the human neuropeptide Y gene that binds Oct-1 and Pbx-1 in GT1-7 neurons.. Biochem Biophys Res Commun.

[32] Frederich RC, Hamann A, Anderson S, Lollmann B, Lowell BB (1995). Leptin levels reflect body lipid content in mice: evidence for diet-induced resistance to leptin action.. Nat Med.

[33] Gao J, Ghibaudi L, van Heek M, Hwa JJ (2002). Characterization of diet-induced obese rats that develop persistent obesity after 6 months of high-fat followed by 1 month of low-fat diet.. Brain Res.

